# Resource requirements for community-based care in rural, deep-rural and peri-urban communities in South Africa: A comparative analysis in 2 South African provinces

**DOI:** 10.1371/journal.pone.0218682

**Published:** 2020-01-30

**Authors:** Donela Besada, Daygan Eagar, Russel Rensburg, Gugu Shabangu, Salamina Hlahane, Emmanuelle Daviaud

**Affiliations:** 1 South African Medical Research Council, Health Systems Unit, Cape Town, South Africa; 2 Rural Health Advocacy Project, Johannesburg, South Africa; 3 Umzinyathi Health District, Kwazulu-Natal, South Africa; 4 Sedibeng Health District, Gauteng, South Africa; University of Oxford, UNITED KINGDOM

## Abstract

**Introduction:**

As South Africa embarks on the implementation of the community health care worker (CHW) 2018 policy, quantifying the resource requirements to effectively manage the programme across different geographical communities is essential. This study was conducted to quantify and compare costs associated with travel and service delivery demands on CHWs between area types in two districts.

**Methods:**

This economic analysis adopted a provider perspective to cost CHW services between January and November 2016. A total of 221 CHWs completed diaries for 10 days to document their activities. Quintile regression and the Kruskall Wallis test were used to test for differences in time and activities across urban and rural sites.

**Results:**

While travel time across rural and urban settings within each district did differ it was not the most significant predictor of differences in time utilization. Time on activities showed more significant differences with overall median time by unit of activity being 15% longer in rural than urban areas in Sedibeng and 10% longer in uMzinyathi respectively. Most CHW time was spent conducting home visits (57% in rural,66% in peri-urban/urban). Median time per home visit in uMzinyathi was 50% longer in deep-rural areas than urban areas and 20% longer in rural than urban areas in Sedibeng. Referrals and number of home visits per capita (0.4 visits in rural and 0.7 in urban/peri-urban areas) were low in both districts. Expenditure on the programme translated to under 4% of PHC expenditure per capita and remains under 5% if despite the new national minimum wage (R3,500/$245).

**Conclusion:**

Because home visits take longer and CHWs spend a lower share of time on visits, a higher number is required in rural and deep rural areas (33% and 66% respectively) than in urban areas. Effective budget planning will therefore need to recognize the different geographical needs.

## Introduction

The South African health system is characterized by a large public sector and smaller private sector as well as Non-Governmental Organizations (NGOs). Although South Africa spends a relatively high percentage (8.6%) of its gross domestic product (GDP) on health, only 44% of total health expenditure (4.1% of GDP) takes place in the public sector, where most of the country’s population (86%) seeks care. This has resulted in sustained inequitable access to health care along socio-economic and geographical lines.[1]

These inequities manifest most starkly in significant disparities in the distribution of Human Resources for Health (HRH), both between public and private sectors and between urban and rural communities. There exists a six-fold difference in the number of people served by nurses and 23-fold difference in the number of people served by a specialist doctors when comparing private and public sector health provision. Despite 43.6% of the population living in rural communities, they are served by 12% of the doctors and 19% of the nurses available in the country; further compounding inequities in access to health care. [2]

As part of an ongoing processes of health system reforms aimed at broadening access to primary health care, Community health workers (CHWs) are being touted as a possible solution to critical human resource shortages and improve health coverage. CHWs have been demonstrated to be an important and cost-effective option in a range of low-and-middle income and underserved contexts where chronic shortages of nurses and doctors and large distances to health facilities have resulted in limited access to essential health services for vulnerable populations.[3] South Africa has a long history of using this health cadre, spanning close to 5 decades. In the 1970s, CHW programmes were implemented by NGOS to address the inequitable distribution of health services resulting from the Apartheid era. Post 1994 however, a focus of primary health care on facility-based services resulted in the collapse of many CHW programmes across the country. A new wave of disease-focused CHWs, supported by NGOs emerged thereafter, predominantly focusing on HIV and TB related treatment and support. While this approach addressed a critical need, it resulted in an underutilization of this cadre with the potential to realize a wider social and health impact by including more programs in CHW scope of practice[4]. The changing burden of disease in South Africa over the past twenty years and reduced mortality due to expanded roll out of antiretroviral therapy (ART), coupled with an ageing population and associated lifestyle diseases has placed considerable pressure on the health system to deliver integrated services.

Presently, CHWs roles have expanded to support the delivery of high priority interventions relating to maternal and child health, HIV, TB and chronic diseases. While they have played an important part in ensuring the success of many of these programmes over the years, there has been significant inconsistency in the management and functioning of CHW programmes across South Africa[5]One of the primary challenges with CHW programmes is that the role of CHWs had never been fully articulated in National or Provincial health policies. The absence of a developed CHW policy has meant that there has been no guidance on CHW qualification requirements, training, employment conditions, or scope of practice [6].

This inconsistency in policy has been made worse by the fact that many CHWs are employed by NGOs and therefore not employees of the department of health; in 2011, the Department of Health was funding 1260 NGOs to pay 41,000 stipends[7], with latest estimates from the Health Systems Trust indicating 54,180 active CHWs. This has meant that not only has their function not been clearly defined but also that their conditions of employment have been determined by the NGOs rather than according to public service regulations. While NGOs would need to meet basic conditions of employment, there is no obligation to employ CHWs on a full-time basis or to provide them with basic benefits, such as a pension, that public sector employees are entitled to receive.

Since 2011 the National Department of Health (NDoH) has been embarking on some ambitious public health reforms, including the development of a policy and piloting of a National Health Insurance System (NHI). Primary Health Care (PHC) re-engineering forms one of the key pillars of the NHI, made up of a four streams approach including contracting private health care practitioners, district-based clinical specialist teams, school health services and ward-based outreach teams (WBOTs).[8] The PHC re-engineering policy has provided general guidance on how WBOT’s should be structured. The policy states that WBOTs should be made up of 6–10 CHWs and their team leader, a professional nurse, as well as environmental health and health promotion practitioners. The policy goes on to note that WBOTs should focus on health promotion and disease prevention activities and work to improve access to primary health care services in South Africa.[9] While the PHC re-engineering strategy encompassed all aspects of WBOT function, a central component of the strategy which was lacking was direction concerning the resourcing of CHWs to ensure consistency in implementation of the programme across differing service delivery contexts.

The lack of detail on CHW scope of practice and programme finance considerations have, to some extent, been addressed in the South African Department of Health’s formalized Policy Framework and Strategy for WBOTs introduced in 2018 (‘the Policy’). An important implementation consideration outlined in the Policy is to ensure that different requirements between urban and rural areas are quantified and costed, to inform various WBOT policy and implementation options and support government’s resource allocation and planning. This study was conducted in anticipation of these policy requirements. Its primary aim is to compare the resource implications (both financial and human resources) to implement the CHW component of the WBOTs within rural and urban communities. The study assesses two different community-based service models implemented in two provinces to highlight potential differences between urban and rural sub-districts. The purpose of this study was therefore to identify possible differences in needs and activities between these types of areas and the resulting implications on resource allocation.

## Methods

### Study design

This economic analysis adopted a provider perspective to determine financial costs borne by the health system to implement the community health care worker programme across different geographical areas in South Africa. Data collection was undertaken between January-November 2016. All rand values were converted to USD (1 USD = 14.3 ZAR)

### Site selection

Gauteng and Kwazulu-Natal provinces in South Africa were purposefully selected due to the availability of functional and complete WBOT teams, in addition to the availability of both urban and rural sites through which both an inter-provincial and intra-provincial comparative analysis could be conducted. Furthermore, the two provinces used different service delivery models that could be described. Discussions with the district health teams facilitated selection of sub-districts and facilities, health posts or WBOT teams.

The Sedibeng district, with a population of 935,831 people covers large poor peri-urban areas including both formal and informal settlements, including farming areas. Sedibeng District spans the entire southern area of the Gauteng Province and is made up of three local municipalities including Emfuleni, Midvaal and Lesedi. Despite Midvaal making up approximately half of Sedibeng's total area, over 80% of the population live in Emfuleni. The eastern part of the district is predominantly agricultural or rural while the urban areas are mainly located in the western regions of the district in Emfuleni, with fewer urban concentrations in the other municipalities. The district's primary health care facilities are clustered in the urban centers, with rural areas being serviced by mobile units. Sedibeng was selected as it was implementing a health post model from which CHWs operated. These health posts are small prefabricated buildings that allow for the nurse team leader to provide support and training to CHWs who convene there every morning before their outreach activities. Furthermore, health posts function as a ‘mini-clinic' providing basic health services.

The UMzinyathi district in KwaZulu-Natal with a population of 513,974 was selected as the second site as it provided different models of community-based services, including CHWs operating mainly from targeted primary health care facilities in addition to WBOT teams working independently. CHWs in KZN are referred to as Community Care Givers but they broadly provide the same services and will be referred to as CHWs in the paper. UMzinyathi district included both urban and deep-rural sub-districts from which a sample could be drawn. uMzinyathi in KZN is a predominantly rural district with one mainly urban sub-district, Endumeni, making up 11% of the population, and three deep-rural sub-districts making up the remainder of the district.

### Classification of sites

The determination of urban/rural sites was based on the National District Health Information System classification system. To further differentiate within rural sites, farm sites were classified as rural while less densely populated sites were characterized as deep-rural. In urban areas, most sites were peri-urban, and urban poor sites in the city centre were included in the categorization urban/peri-urban.

As community-based care services (CBS) are directed more specifically to poorer areas, the term ‘urban' in the study reflects, in fact, a mix of urban impoverished communities, urban informal settlements and peri-urban settlements. In uMzinyathi the term ‘rural' refers to deep-rural settlements while in Sedibeng the term ‘rural' to regrouped farm worker settlements on farms.

### Data collection

To measure the time CHWs spend on travel and tasks, 11 and 74 WBOT teams in both Sedibeng and uMzinyathi respectively covering different types of geographical areas were randomly selected to complete diaries for two consecutive weeks (10 days). The methodology for the construction of the diaries was based on a similar study conducted in Ethiopia by Mangham et al.[10] to determine how CHWs spend their time. Piloting and adaptations of the tool were made for this study according to the South African setting and CHW scope of practice.

**Fig 1:**
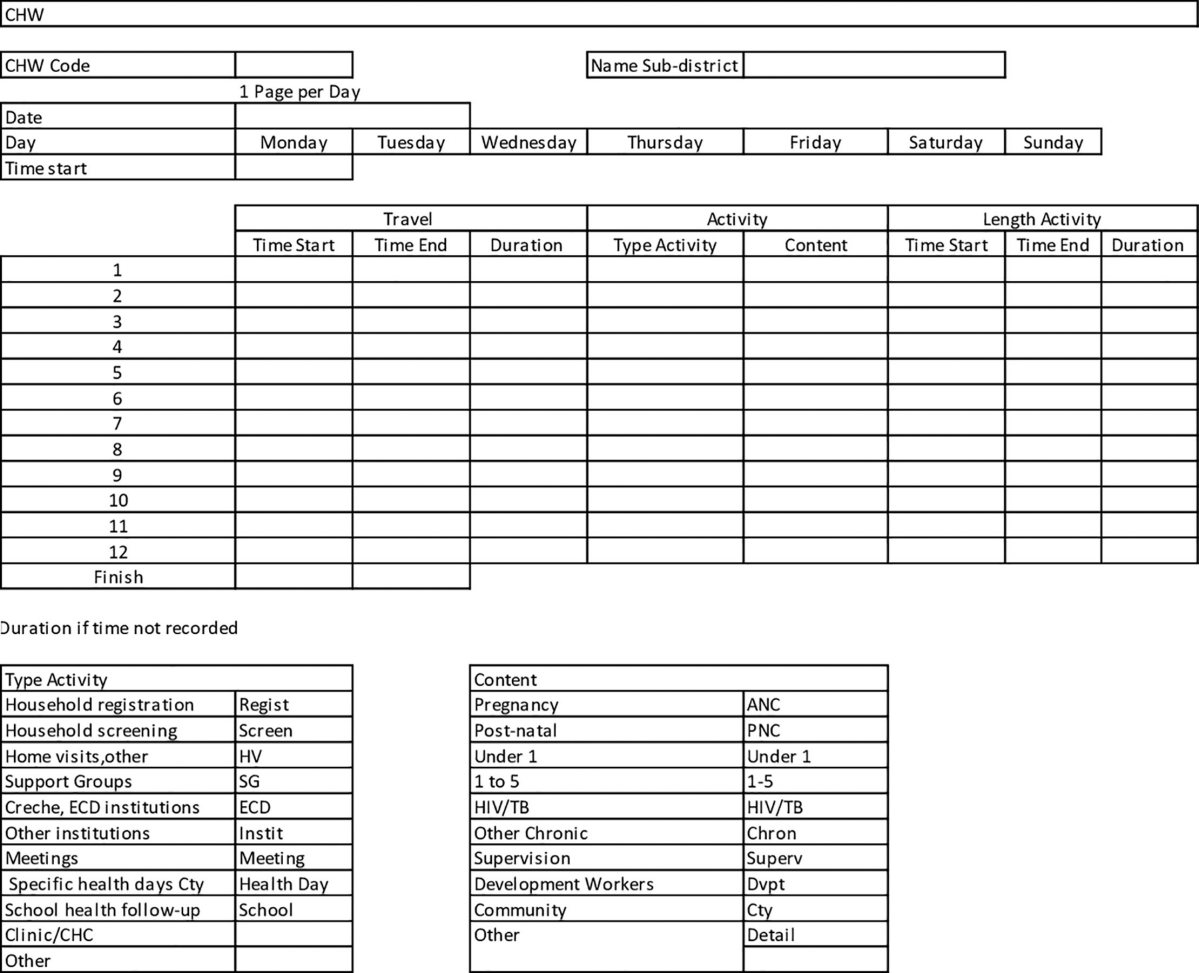
CHW diary.

The diaries allowed for day to day collection of CHW activities including the time it took to travel and to conduct their day-to-day activities, the types of activities, disease areas being addressed and the type of recipients they saw (Fig 1). The 110 CHWs in uMzinyathi and 111 in Sedibeng were trained in the use of the tool in teams consisting of 7 to 10 CHWs in Sedibeng, and 1–2 CHWs per identified facility in uMzinyathi. As a result, a far larger number of teams were sampled in uMzinyathi to recruit a comparable number of CHWs. The start of data collection was staggered as the training took place. After the initial two days of diary filling and at the end of the week each team was revisited by a researcher to assess the accuracy of the data being inputted and redress possible mistakes.

### Data analysis

The data was inputted into Excel and exported into Stata v.13 for analysis. As the data was not normally distributed, significance testing on the median travel and activity times were conducted through quantile regression for individual activities while the Kruskall Wallis test was used to test overall significance in travel and activity time between different services across urban and rural sites. Significance testing for the proportion of share of time per activity, as well as the content, conditions and recipients during home visits used the Test of Proportions.

Data was extracted from the District Health Information System (DHIS) to determine population access to services in each district. For each primary health care facility (clinic, community day centre, community health centre) with WBOT teams, the population per facility, the type of facility, the number of facility headcounts, and the number of home visit headcounts were extracted. For each type of site, the average number of PHC facility visits per capita and the number of home visits headcounts per capita for the period April 2015 to March 2016 were then calculated. The Kruskal-Wallis test was used to assess any significant differences.

In Sedibeng half of the WBOT teams are based at health posts and therefore a share of the health post and professional nurse costs placed there were allocated to community-based services.

Costs are presented in 2 categories: set-up and recurrent costs. Capital costs were annualized using straight depreciation (cost/number life years).

#### Set-up costs

- Health posts: While some health posts were donated, new health posts are now being bought by the district. Thirty five percent of health post costs were allocated to CBS. The purchase cost of the health post and its equipment was annualized over 15 years.
- Training: In 2015, CHWs received two training sessions, each lasting 2-weeks. Training is organised by the provinces and costs were not available. We thus extracted the cost of training from the investment case carried out in 2015[11] indicating a cost of R6,000 ($420) per person for the four weeks training. In uMzinyathi, supervisors received training as part of the ongoing monthly in-service training, and community health facilitators have a short supervisory training. Formal training does not take place every year, therefore training was annualized over a 5-year period.
- Kits: CHWs received kits which varied between the two districts. On advice of the district, we allocated two life years to the kits in Sedibeng and uMzinyathi. In both districts, CHWs do not get cell phones or airtime.

#### Recurrent costs

- Staffing: Salary/Stipends for the end of 2015 were applied for the analysis. In Sedibeng dedicated nurse supervisors in health posts were retired nurses with a pension. They were paid a top-up of 13% of their salary with no benefits. An average of 55% of the professional nurse's time were allocated to the supervision of WBOTs. CHWs were paid a monthly stipend (R2,500 ($175) in Sedibeng, R1,800($126) in UMzinyathi).
- Kits: We added the annual cost of supplies for kit replenishment. In uMzinyathi, vitamin A and D, the only medicine products provided by CHWs, are provided by the Phila Mtwana centre (nutrition centre) and are not included in the kit replenishment costs.
- Health posts maintenance: We attributed 35% of the annual health post maintenance cost in Sedibeng
- Transport: CHWs walk to houses, and no transport refund is available for meetings. In uMzinyathi, a dedicated car is available for the community health facilitator who transports the CHW supervisors as required.

We then calculated costs as annualized set-up costs plus recurrent costs. We present cost for the district, cost per capita total population and cost per CHW. With the on-going discussions on stipend level for CHWs, and recent decision to increase their stipend to the minimum wage, we modelled the implications of this decision on the health budget. We thus assumed a monthly stipend of R3,500($245) and calculated the increase in district expenditure and WBOTs expenditure per capita for the existing number of CHWs.

To determine the adequacy of the observed level of activity and assess the number of CHWs required for a given population by type of site, the distribution of CHW activity from the diaries were compared with those suggested in the 2012 report to the NDoH, that identified staffing norms to inform short and medium term planning for South Africa’s PHC re-engineering plan.[12] This report models the number and type of home visits for a population based on the Stats SA 2016 demographic structure of the population[13] and its burden of disease, extracted from the DHIS and the District Health Barometer published by the Health Systems Trust[14]. The data was standardized per 100,000 total population. The number of home visits which would be required with 70% coverage of CHWs was calculated, because of the unlikelihood that South Africa would achieve 100% coverage in the infancy of the programme and as a result of fiscal constraints. This was then compared with the number of home visits per capita in areas with WBOTs teams, still assuming 70% coverage. For each district, two scenarios were explored including one in which the whole population was urban/peri-urban and the latter in which the whole population was rural or deep rural.

We assessed the time required to cover the home visits and other activities for each scenario and each district by using the median time of combined travel and length of activity per type of home visit from the analysis of the CHWs diaries and applying the number of home visits modelled for 100 000 population to calculate home visit time required. We then applied the observed share of CHW time spent on home visits to calculate total CHW time required for home visits and other activities for the 100 000 population. From the total CHW time required we modelled the number of CHWs required for each scenario separately for Sedibeng and uMzinyathi, as well as the support/supervision structures, applying the organizational policies of each district. CHWs work 30 hours a week in Sedibeng, but 40 hours in Umzinyathi, while CHWS work in pairs in Sedibeng for security reasons but on their own in Umzinyathi. We then applied the findings relating to the difference in number of CHWS required by type of site to calculate the number of households a CHW should cover by type of site. We used as the base the number of 250 households per CHW recommended in government guidelines and applied this number to urban/peri-urban areas.

### Ethical considerations

Ethics approval for the study was provided from the University of Witswaterstrand Human Research Ethics Committee: H15/06/23 as well as approval obtained from the Kwazulu-Natal Department of health: HRKM: Ref 191/15 NHRD: ref: KZ_2015RP2_671 and Sedibeng Department of Health. Verbal informed consent was obtained from all Community Health Workers who filled the diaries.

## Results

### Median travel and activity time

A combination of rural (farms) and peri-urban sites were sampled in Sedibeng for the completion of diaries; peri-urban sites represented 68% of the sample while rural sites represented the remaining 32%. A total of 111 CHWs were sampled. The number of CHWs per site ranged from 4–5 per health post up to 25 CHWS when they were attached to PHC facilities. Rural sites in uMzinyathi refer to deep-rural areas. Deep rural sites represented 77% of the sample, peri-urban sites the remaining 23%. A total of 110 CHWs were sampled.

Table 1 outlines the median travel time and time spent on the range of activities undertaken by CHWs in Sedibeng and UmZinyathi as well as the share of time spent on the different activities. In Sedibeng, different travel and activity times on a range of CHW activities were identified between peri-urban and rural sites.

**Table 1:** Travel time and time spent on activities in Sedibeng and uMzinyathi as well as share of time per activity.

**Health District: Sedibeng**						
	**Travel and time spent on activities**				**Share of time per activity**	
	Median Travel Time (Minutes)		Median Activity Time (Minutes)		Peri-urban	Rural
Activity	Peri-urban	Rural	Peri-urban	Rural		
Household Registration	15	15	19*	24*	66%*	57%*
Household Screening	15	17	15*	23*		
Home Visits (other)	15	15	20	20		
Health Post/Clinic/CHC	15*	30*	25	30	23%*	29%*
Support Groups	28	16	10	34	0%	1%
Creche, ECD inst	20		15		0%	1%
Other institution	10	25	10	10	0%	0%
Health days	15	20	20	15	0%	0%
School Health F/U	18	10	53	21	0%	0%
Meeting	30	165	330	225	1%	3%
Other	17*	9*	24*	30*	8%	8%
**Health District: Umzinyathi**						
	**Travel and time spent on activities**				**Share of time per activity**	
	Median Travel Time (Minutes)		Median Activity Time (Minutes)		Peri-urban	Rural
Activity	Peri-urban	Rural	Peri-urban	Rural		
Home visits	15*	20*	40*	60*	55%*	49%*
Clinic/CHC/Mtg w supervisor	25*	30*	120	115	18%*	**13%**
Phila Mtwana	30	30	360*	425*	15%*	16%*
Creches and other institutions	27	23	50	73	1%	2%
Campaigns	20	20	150	235	2%	3%
Meeting with community/other	20	30	40	100	3%*	4%*
War Room	30	35	122	188	2%	2%
Training	35	30	51*	120*	2%	5%
Support Groups	30	30	55	95	1%	1%
Other	16	15	43	40	1%	5%

Median values (in minutes) are reported here for comparison purposes as the data was not normally distributed. Significance testing on the median travel and activity times were conducted through quantile regression for individual activities. Significance testing between the proportions of time spent on activities across geographical areas was conducted through a test of proportions.

CHC: community health centre, ECD: Early childhood development, F/U: follow up, Mtg: meeting

*Values in bold in the table indicates that differences in medians were statistically significant.

CHWs in rural sites in Sedibeng spent 7% longer travelling than those in peri-urban sites and 15% longer on their activities. CHWs travelling to the clinic in rural sites spent a median of 30 minutes compared to 15 minutes to reach the health post, clinic or CHC in the peri-urban areas. It was also found that CHWs in rural areas spend 26% longer on household registration and 53% longer on household screening during home visits. Travel time represented over 40% of the total share of CHW time.

Overall, both travel time and activity time recorded were significantly different between peri-urban and deep-rural sites in Umzinyathi. CHW travel time for home visits was 33% longer in deep-rural areas than in peri-urban areas, and it also took 20% longer to travel to clinics in deep-rural areas. With regard to activity time in Umzinyathi, CHWs in deep-rural areas spend 50% longer seeing clients during home visits, and 18% longer within Phila Mtwana PHC centers. These centres represent a simple structure where basic health promotion and therapeutic services, including early childhood development, can be accessed by the community. CHWs in deep-rural areas spend slightly less time in the clinic as compared to CHWs in peri-urban sites. Travel time made up a much lower share of the total time in uMzinyathi than it did in Sedibeng, reflecting approximately 20% of total CHW, with the remaining time spent conducting activities.

Analysis of the share of CHW’s time per broad type of activity in Sedibeng after combining travel and activity time shows that home visits took up the majority of CHW time in both rural and peri-urban areas. These home visits reflect 57% and 66% of their total recorded time. CHWs in rural areas spent 29% of their time in clinics compared to 23% in either health posts or clinics in peri-urban areas. Meeting with supervisors in the facilities is included in facility time. CHWs are expected to meet either in the health post or clinic in the morning before beginning home visits and daily activities and come back at the end of the day. If CHWs work directly out of the health post, clinic visits are required for training activities or to pick up the chronic medication for home deliveries.

While the share of time spent on different activities in uMzinyathi, after combining travel and activity time, varied little between peri-urban and deep-rural areas, significant differences could be observed with CHWs in peri-urban areas spending a larger proportion of their time on home visits (55% vs. 49%) and in the clinics (17% vs. 11%).

### Content of home visits and referrals

While screening and health education were often involved in home visits in Sedibeng (Table 2), bringing medicines to homes took place in 28% of home visits in peri-urban areas but in only 18% in rural areas. The large proportion of time spent bringing drugs to a patient in the home is because in Emfuleni, Sedibeng’s peri-urban area, CHWS deliver pre-packaged chronic medication to elderly patients in the home as part of what is known as the ‘Kgatelopele programme’ to improve patient adherence for diabetes and hypertensive drugs. Tracing defaulters was the reason for about a quarter of home visits in rural areas but only 4% in peri-urban areas. Home-based care (HBC), consisting of CHWs dressing wounds and helping wash patients, was twice as common in rural than peri-urban areas. Few home visits translated into referrals by the CHWs in both rural and peri-urban areas (under 4%).

**Table 2:** Content of home visits and referrals.

**Health District: Sedibeng**		
*Activity*	Peri-urban	Rural
Screening	40%*	30%*
Health educ	30%*	19%*
Bring meds	28%*	18%*
Tracing defaulters	4%*	26%*
HBC	3%*	7%*
Referral	4%	3%
DOTS	3%	2%
Other	4%*	7%*
**Health District: Umzinyathi**		
*Activity*	Peri-urban	Rural
Screening	26%	23%
Health Education	34%*	49%*
Bring medicine to patient	12%*	7%*
Referral	2%*	12%*
Child Health	8%*	12%*
HBC	9%	8%
Tracing defaulters	5%	6%
DOTS	5%	4%
Other	3%	5%

Significance testing between the content of the home visits and referrals across geographical areas was conducted through a test of proportions. DOTS: Directly Observed Treatment, Short Course, Educ: Education HBC: Home based Care

*Values in **bold** in the table indicates that differences in proprotions were statistically significant.

Most home visits involved heath education and/or screening in both types of sub-districts in Umzinyathi, with health education taking place more often in deep-rural areas (49% vs. 34%). Bringing medicines to patients was not a frequent occurrence, although it was more frequent in peri-urban than deep-rural areas (12% vs. 7%) while the reverse was true for home visits related to child health (8% vs.12%). No difference could be observed between areas with regard to the frequency of defaulter tracing; CHWs in both sites only traced defaulters approximately 5% of the time. Referral rates were low in both areas but reflected 12% of home visits in deep-rural areas, substantially higher than in peri-urban areas (2%).

### Conditions

The conditions addressed during the home visits in Sedibeng (Table 3) showed different patterns between peri-urban and rural areas, with a larger share of home visits for mothers and children under 5, family planning, and chronic conditions in peri-urban areas. In rural areas, TB-related visits were almost twice as frequent as in peri-urban areas, in addition to nutrition-related visits being more frequent than in peri-urban areas. No difference could be observed in HIV-related visits.

**Table 3:** Conditions addressed during home visits.

	Sedibeng		Umzinyathi	
Service Type	Peri-urban	Rural	Peri-urban	Rural
Mother/child	7%*	4%*	13%	14%
HIV	20%	21%	11%*	18%*
TB	20%*	38%*	11%	14%
Chronic	45%*	26%*	28%*	19%*
Nutrition	3%*	5%*	14%*	19%*
FP	12%*	8%*	20%	23%
Other	5%	5%	5%*	10%*
Unknown	4%	3%	2%	1%

Significance testing for the conditions addressed during home visits was conducted through a test of proportions. FP: Family Planning, Mother and child relates to antenatal and postnatal care, immunisations, etc. Chronic: relates to conditions including hypertension and diabetes

*Values in bold in the table indicates that differences in proportions were statistically significant.

Overall the type of conditions addressed during home visits in uMzinyathi showed limited differences between peri-urban and deep-rural areas, except for family planning, which presented a larger share of home visits in deep-rural areas (18% vs. 11%) and TB taking up a larger share in peri-urban areas (28% vs. 19%).

### Recipients

For most home visits CHWs recorded seeing only one person (diaries allowed for the recording of up to 3 recipients), with the average number of recipients standing at 1.1 per home visit in both districts (Table 4). A larger share of home visits to pregnant women and infants were observed in peri-urban than rural areas in Sedibeng. Furthermore, while more adults are visited in rural than peri-urban areas, there were fewer visits with elderly recipients in rural areas.

**Table 4:** Recipients seen during home visits.

	Sedibeng		Umzinyathi	
Service Recipient	Peri-urban	Rural	Peri-urban	Rural
Preg woman	4%*	2%*	3%*	6%*
Post Natal	**2%**	**1%**	3%*	6%*
Infant	**2%**	**1%**	3%	4%
1–5 yrs	5%	4%	9%*	19%*
5-18yrs	5%*	10%*	4%*	11%*
Adult	48%*	59%*	49%	47%
Elderly	30%*	23%*	7%	9%
Family	12%	14%	12%	14%
Comm	1%	0%	5%*	2%*
Unknown	1%	3%	4%*	0%*

Significance testing for the recipients seen during home visits was conducted through a test of proportions. Comm: community, Elderly: >60; Adult: 20–59 years

*Values in bold in the table indicates that differences in proportions were statistically significant.

Mothers and children under 5 represented 18% of recipients in peri-urban areas and a significantly higher share in deep-rural areas amounting to 34% in UmZinyathi. Most recipients in both deep rural and urban sites were adults under 60. Elderly people represented a small share of recipients, largely due to the young demographic structure of the district.

### Cost of WBOTs in Sedibeng

In Sedibeng, half of the 44 WBOT teams are based in health posts. The costs are presented separately for health-post based teams and facility-based teams (Table 5). The purchase cost of a health post and its equipment amounted to just under R530,000($37,063) per unit. CHW kits cost R2,495 per kit and are expected to last for two years, excluding the replenishment of supplies. Monthly stipends/salary package stood at R2,500($175) per CHW, R20,937($1464) per staff nurse and R3,869($271) per retired nurse on a full-time basis. On average, professional nurses spent 55% of a full-time equivalent (FTE) on WBOTs, the remainder of their time being spent on consultations or other health post/facilities duties. No additional supplies (uniform, cell phone and airtime) are given to CHWs or their supervisors. The combined cost to the district of the teams based in health posts and facilities amounts to just over R44 million ($3,076,923), or R47($3.3) per capita total population, equivalent to 3.9% of the district PHC expenditure per capita. Expressed in cost per CHW, it amounts to about R52,000($3636) per CHW. Management/Supervision (Assistant director, professional nurses and staff nurses) represent 28% of the annualized expenditure and training represents 2.3% of expenditure.

**Table 5:** WBOTs cost in Sedibeng.

Sedibeng						
HEALTH POSTS	Unit Cost	Quantity	Life Years	% for WBOTs	Annualised District Cost	Annualised Cost per CHW
Set-up						
Infrastructure	490,000	22	15	35%	251,533	593
Equipment: Health Post	39,200	22	15	35%	20,123	47
Equipment: kits	2,495	424	2	100%	528,940	1,248
Training: CHWs	6,000	424	5	100%	508,800	1,200
**Recurrent**						
Salaries/Stipends						
Asst Dr	426,000	1	1	50%	213,000	502
Professional Nurses	46,423	22	1	55%	561,721	1,325
Staff Nurses	251,244	22	1	100%	5,527,375	13,036
CHW WBOTs	30,000	424	1	100%	12,720,000	30,000
Health Post Maintenance	10,000	22	1	100%	220,000	519
Kit replenishment	250	424	1	100%	106,000	250
**Overheads@8%**					**1,652,599**	**3,898**
**Total**					**22,310,091**	**52,618**
**FACILITIES**	**Unit Cost**	**Quantity**	**Life Years**	**% for WBOTs**	**Annualised District Cost**	**Annualised Cost per CHW**
**Set-up**						
Equipment: kits	2,495	424	2	100%	528,940	1,248
Training CHWs	6,000	424	5	100%	508,800	1,200
**Recurrent**						
Salaries/Stipends						
*Asst Dr*	426,000	1	1	50%	213,000	502
*Professional Nurses*	46,423	22	1	55%	561,718	1,325
*Staff Nurses*	251,244	22	1	100%	5,527,375	13,036
*CHW WBOTs*	30,000	424	1	100%	12,720,000	30,000
Kit replenishment	250	424		100%	106,000	250
**Overheads@8%**					**1,613,267**	**3,805**
**Total**					**21,779,100**	**51,366**

### WBOTs Cost in uMzinyathi

Table 6 below outlines the cost of the WBOT programme in uMzinyathi. In uMzinyathi, CHWs receive a monthly stipend of R1,800($126) while their supervisors receive R2,300($161). Community health facilitators, who then support the supervisors, are made up of staff nurses or nursing assistants. We pegged their salary to a mid-point nursing assistant at R177,660(12,424). The district CHW co-coordinator is an assistant director. The annualised cost for the district, excluding offices and support for the district CHW coordinator, amounted to just under R14 million($979,021), translating to R28($2) per capita total population or 2.4% of PHC expenditure per capita. Expressed in cost per CHW it amounted to R29,948($2094) per CHW. Management/supervision constituted 16.7% of the annualized expenditure and training made up 5.8% of expenditure.

**Table 6:** WBOTs cost in uMzinyathi.

Umzinyathi					
	Unit Cost	Quantity	Life Years	Annualised District Cost	Annualised Cost per CHW
**Set-up**					
Training/ Supervision	2000	6	5	2400	5
Training CHWs	6000	481	5	577200	1200
Car for community health facilitators	350,000	6	5	420000	873
**Equipment**					
Kit	510	481	2	122655	255
jacket+umbrella+coolerbox	477	481	1	229466	477
**Recurrent**					
Salaries/stipend					
*District CHW co-ordinator*	426,000	1	1	426,000	886
*Community health facilitator*	177,660	6	1	1,065,957	2216
*CHW supervisor*	27,600	16	1	441,600	918
*CHW*	21,600	481	1	10,389,600	21,600
Kit replenishment	671	481	1	322,751	671
Transport supervisors	101839	4	1	407,356	847
**Total**				**14,404,985**	**29,948**

### Utilization in peri-urban and rural areas

The pattern of utilization of services (Table 7) was different between the two districts, with uMzinyathi showing a higher level of clinic/CHC utilization (3.1 visits per capita per year) than Sedibeng (just under 2). In Sedibeng, the clinic headcount per capita/year was significantly higher in theperi-urban than the rural setting, as was the number of home visits per capita, based on areas with functional WBOTs (1 home visit per capita in peri-urban and 0.4 in rural areas). In uMzinyathi, the number of home visits per capita stood at 0.7 in peri-urban and 0.4 in deep-rural areas.

**Table 7:** Health care utilization in Sedibeng and uMzinyathi.

	Sedibeng		UmZinyathi	
	Peri-Urban	Rural	Peri-Urban	Rural
Headcount per capita/Year (clinic)	2	1.7	3.2	3.1
Home visits per capita/year (70% coverage)	1	0.4	0.7	0.4
Total contacts with PHC/capita/year	2.7	2	3.7	3.4

The expected number of home visits per capita, after applying the demographic structure and burden of disease of each district, for an average 70% coverage, would amount to an average of 1.2 home visits per capita per year in Sedibeng and 1.5 in uMzinyathi.

### Number of CHWs required per type of geographical area

Applying time on travel and activity time per type of activity and type of home visits from the data presented earlier, the number of CHWs required per type of district was calculated. The number of CHWs required would be so that there were an equal number of home visits and population coverage and not necessarily the same package of care or specific services (activities) during those home visits.

In Sedibeng, CHWs spent 66% of their time on home visits (travel + time in homes) in peri-urban and 57% in rural areas. If the district was entirely peri-urban, and with CHWs working in pairs, 334 (168x2) CHWs would be required to cover home visits and other activities per 100,000 population. If the district was entirely rural, with people living on farms, 446 (223x2) CHWs would be required, 33% more than in an peri-urban district. If CHWs did not work in pairs, this proportional difference would remain the same. (Table 8) There are currently 88.5 CHWs per 100,000 population in Sedibeng. In uMzinyathi, CHWs spent 55% of their time on home visits in peri-urban and 49% in deep-rural areas. Unlike in Sedibeng, CHWs do not work in pairs. To cover home visits and other activities for a total population of 100,000 people, 137 CHWs would be required if the population was all living in peri-urban areas. If 100% of the population were living in deep rural areas 221 CHWs would be required, 62% more than if the district was all peri-urban. There are currently 87 CHWs per 100,000 population in uMzinyathi.

**Table 8:** CHWS required for 100,000 population per type of site in Sedibeng.

	Median Time Per Visit (minutes)			
	**Sedibeng**		**Umzinyathi**	
Service	Peri-Urban	Rural	Peri-Urban	Rural
Registration	35	40	85	75
Screening	35	40	35	90
Mother & under 5	36	40	71	110
HIV/TB	35	35	61	90
Chronic	35	45	72	89
Other	39	50	110	120
	**Sedibeng**		**Umzinyathi**	
Total Time (Hours) per Service per 100,000 population	Peri-Urban	Rural	Peri-Urban	Rural
Registration	1,295	1,480	5,441	4,801
Screening	1,295	1,480	7,169	18,434
Mother & under 5	21,419	23,799	23,339	36,159
HIV/TB	18,579	18,579	39,974	58,978
Chronic	24,022	30,885	44,589	55,117
Other	13	17	38	42
Total Time (Hours) for all Services per 100,000 population	66,623	76,240	120,549	173,530
Proportion of CHW Time spent on Home Visits	66%	57%	55%	49%
Total CHW days on duty, per year	200	200	200	200
Total CHW hours on duty, per day	6	6	8	8
Total CHW hours available for Home Visits, per year	792	684	880	784
Total CHWs per Home Visit	2	2	1	1
Total CHW required	168	223	137	221
Difference in CHWs Required	33% more CHWs required in rural site		62% more CHWs required in rural site	

### Households per CHW

According to the government guidelines a CHW should cover 250 households[15]. Using this base for peri-urban areas, the number of households per CHW in rural areas should be 33% lower or 169 households, and 62% lower in deep-rural areas or 96 households per CHW (Table 9).

**Table 9:** Number households per CHW per type of site.

Type of area	Ratio to Peri-Urban sites	# households per CHW
Peri-urban	1	250
Rural	-33%	169
Deep-rural	-62%	96

### Sensitivity analysis

Increasing the CHW stipend to R3,500 ($245) a month would increase the Sedibeng district expenditure by 25% and the WBOTs expenditure per capita from R47($3.3) to R59($4.1). WBOTs expenditure would still represent under 5% of PHC expenditure per capita. In the less resourced uMzinyathi district, the stipend would almost double, translating to an increase of 71% in district expenditure on the WBOTs. The WBOTs expenditure per capita would move from R28(2) to R48($3.4) but would still represent only 4% of PHC expenditure per capita. (Table 10)

**Table 10:** Financial impact of CHW stipend increase to national minimum wage.

	Current	Modelled to Minimum Wage
**Sedibeng**		
CHW stipend per month	2500	3500
% increase in stipend from current to modelled wage	40%	
% increase in district expenditure from current to modelled wage	25%	
WBOTs expenditure per capita	47	58
PHC expenditure per capita	1200	1200
WBOTs as % PHC expenditure	4%	5%
**Umzinyathi**		
CHW stipend/month	1800	3500
% increase in stipend from current to modelled wage	94%	
% increase in district expenditure from current to modelled wage	71%	
WBOTs expenditure per capita	28	48
PHC expenditure per capita	1150	1150
WBOTs as % PHC expenditure	2%	4%

## Discussion

This is one of the first economic analysis of activities and resource requirements across different geographical areas of the newly scaled up WBOTs in South Africa. There has been significant discourse and progress around the scope of practice and training needs of community health workers with the formalization of the policy in South Africa; an area that has remained relatively unexplored however is how CBS requirements differ between peri-urban, rural and deep-rural areas, for a type of service focusing largely on home visits. We have shown that current expenditure on the programme represents under 4% of total primary health expenditure, and even with the increased minimum wage, expenditure will still fall under 5% in both districts. The study further highlights the need for increased resourcing in rural communities to ensure improve equity in health service coverage.

Annualized costs expressed as costs per CHW amounted to approximately R52,000($3636) in Sedibeng and R29,948(2094) in uMzinyathi. Cost differences between districts can be explained by the higher CHW stipend in Sedibeng (R2,500($175) per month compared to R1,800($126) in uMzinyathi) and the different supervision/management structure with supervisors in Sedibeng being retired nurses and staff nurses while in uMzinyathi they are ‘higher level-CHWs' with a stipend of R2,300($161). Retired nurses received a low salary, made up of a top up percentage of their pension, amounting to R3,869($271) a month for a full-time nurse, without added benefits. While this represents an affordable solution to provide supervision to CHWs given their skills and limited number of practicing professional nurses available for this task, it is challenging, given the average age to expect the retired nurses to walk significant distances to houses to provide on-site supervision. The new policy therefore considers enrolled nurses to provide this supervision role but will require professional nurses to provide them with support.

Travel time was expected to be the main difference between peri-urban, rural and deep-rural areas. Interestingly in Sedibeng median travel time was overall only marginally longer in rural than peri-urban areas. This could likely be explained by the fact that on farms families of agricultural workers are housed in pseudo-settlements, and therefore living close to one another. Surprisingly, the same observation regarding travel time is made in uMzinyathi; however, differences are observed with regards to the activities conducted. Overall median time on activities was 15% longer in rural than peri-urban areas in Sedibeng and 10% longer in uMzinyathi. The median time per home visit in uMzinyathi was 50% longer in deep-rural areas as compared to peri-urban areas and 20% longer in rural areas of Sedibeng. All these differences were statistically significant, and further research is needed to understand the reasons behind these differences. These differences may be attributed to a wider range of needs in rural and deep-rural areas compared to peri-urban areas during home visits or alternatively, the nature of the social interactions that may take place in the homes. Other regional studies aiming to identify how CHWs spend their time, particularly in rural settings, showed that a large proportion of time is spent providing promotion and prevention services, where communities face a number of health issues needing daily clarification including maternal and under five education and other health promotion. [16] [17] In many cases, CHWs invest more time in such settings, even when compared to facility based staff in rural communities.[18]This difference however impact the number of home visits CHWs in each of the areas can perform.

Reasons for home visits varied across both across districts and within the different geographical communities of each district, partly because of the different services being delivered by CHWs in UmZinyathi and Sedibeng in addition to the different community needs as a result of the demographic profile of the sub-districts. Of concern were the low referral rates in both districts, 4% in Sedibeng, and only as high as 12% in UmZinyathi’s deep rural areas. These low referral rate raises concerns about the quality of screening conducted by the CHWs during the home visits and perhaps the quality of their training and supervision. The presence of dedicated supervisors who may provide on-site support would likely elicit higher case finding and higher rates of referral. Furthermore, dedicated supervisors may also improve the efficiency of CHWs and translate into a higher number of home visits per day, however this will need to be validated. A recently conducted study of focus group discussions of CHWs working in a sub-district in the Eastern Cape revealed similar challenges with infrequent on-site supervision [19] despite expectations that supervisors would meet with CHWs weekly and perform field visits at least once a month. Under 50% of the sampled CHWs noted that they met with their supervisor once a month or less, with most of these visits being clinic-based. Another study aiming to profile current CHWs based in KZN[20] identified poor communication between them and facility-based nurses in the referral of clients, leading to poor job satisfaction among CHWs and an understanding of ongoing health services provided to clients. A study[21] to explore the role of on-site supervision of CHWs in South Africa found that those supervised by senior nurses were more motivated and performed a greater range of tasks, and those that were clinic-based were better integrated in the health system and therefore more able to ensure continuity of care. In contrast, teams supervised by junior supervisors, or based in the community, had less engagement with clinic staff, and were less able to ensure necessary care for patients, resulting in lower levels of trust from clients. These considerations will have to be explored as South Africa formalized the programme.

Since CHWs spend a longer time traveling to and within homes during visits in addition to spending a lower share of total time on home visits in rural areas, a higher number of CHWs will be required in rural/deep-rural areas than peri-urban areas to achieve the same level of coverage. The study found that a rural (farming) district would need 33% more CHWs than an peri-urban district in Sedibeng, while in uMzinyathi, a deep-rural district would require 62% more CHWs than an peri-urban district, assuming consistency in the current activities and levels of care. These numbers may change based on shifts in the kinds of services being delivered however. This demonstrates that there are geographical implications on the number of recipients CHWs can cater to daily and should be considered when developing policy and allocating resources towards the programme. According to this estimate and based on the current policy for an average of 250 households per CHW in peri-urban area, a CHW in a rural area would be expected to cover 169 households and 96 households in deep-rural areas. Although the cost per CHW is the same in rural and peri-urban areas, the increased number of CHWs required in rural and deep-rural areas means that budget allocations should be higher per capita in rural than peri-urban areas.

Drawing on previous modelling work conducted by the South African Medical Research Council[12], the number of home visits per capita falls below what would be expected per year based on the demographic structure and burden of disease of the country, suggesting an underutilization of this level of service. South Africa has recognized the importance of community-based services and has identified it as a critical component of strengthened primary health care services. Community based services are envisioned to be complementary to facility-based care and serve to increase population demand and access to health services. The scope of services that CHWs provide address the myriad of health needs of the South African population, ranging from maternal and child care to the quadruple burden of disease in adults including HIV, TB and the range of chronic conditions including diabetes and hypertension. The move towards the institutionalization of CBS within South Africa’s PHC Re-engineering plans has facilitated a movement at national and provincial level towards the development of policies and formal institutionalization of CHWs. A systematic review of the determinants of success in scaling up and sustaining CHW programmes in low-and middle-income countries (LMIC)[22] has identified a range of enabling factors and barriers to scale up of CHWs including: (1) CHW program design and management, (2) community fit, and (3) integration with the broader environment. Understanding how these factors play out in different geographical communities is essential for addressing different community needs.

Some limitations to the study need to be noted. Firstly, the basis of the geographical classification of sites from the DHIS is unclear and further work on clarifying Urban, peri-urban and rural classifications is needed for accurate comparisons. Secondly, the use of self-completed diaries enables a significant number of CHWs to fill diaries but raises the issue of reliability of the data. The alternative approach is applying a time and motion study which, due to its costs, would cover a significantly smaller number of observations and carries the limitation of the Hawthorne effect[23] through which observed participants may modify their actions, knowing they are observed. Furthermore, while the study did attempt to collect cost data related to out-of-pocket transport costs incurred by CHWs to come to work, the quality of the data was insufficient to report. As a result, this study does not cover costs to the CHWs. Understanding these costs are however integral to effective management and planning for this layer of service delivery. A further area not explored in the study, but critical to the effective functioning of the programme, relates to support and supervision for CHWs. Whilst Sedibeng has recently appointed staff nurses as dedicated WBOT team leaders to complement and in some places replace retired professional nurses, in uMzinyathi immediate support/supervision is carried out by higher level CHWs, raising concerns related to the extent of their clinical skills. Further research should be done to assess the impact of the different range of clinical skills of CHW supervisors on their referral rates and accuracy of diagnosis. It is also important to note that the selection of Gauteng and KZN as the provincial sites is due to the fact that the deployment of outreach teams is comparatively well-developed, and therefore conditions in other provinces may be more inequitable.

## Conclusion

Peri-urban, rural and deep-rural areas have different needs concerning community-based services, and such differences must be considered in resource allocation decisions. The study demonstrates that rural and deep-rural areas need more CHWs than peri-urban areas to provide effective coverage. Beyond the different geographical needs, overall, community-based services are extremely under-resourced; even when increasing CHW stipends to the national minimum wage, CBS services would represent under 5% of PHC expenditure per capita/ Increased resourcing towards supervision for CHWs is necessary to ensure the availability of dedicated supervisors with some level of clinical skills (ENs) who can support the work of the community health workers.

## Supporting information

S1 Dataset An anonymized dataset generated and analysed for this study. (XLSX)
